# Addition of bevacizumab to EGFR tyrosine kinase inhibitors in advanced NSCLC: an updated systematic review and meta-analysis

**DOI:** 10.3389/fphar.2023.1238579

**Published:** 2024-01-09

**Authors:** Haosheng Zheng, Xianyu Qin, Yuzhen Zheng, Xingping Yang, Jian Tan, Weijie Cai, Shiyun He, Hongying Liao

**Affiliations:** ^1^ Department of Thoracic Surgery, The Sixth Affiliated Hospital, Sun Yat-sen University, Guangzhou, China

**Keywords:** EGFR, NSCLC, bevacizumab, EGFR-TKI, combination therapy

## Abstract

**Background:** The synergistic effects of antiangiogenic inhibitor bevacizumab and epidermal growth factor receptor-tyrosine kinase inhibitors (EGFR-TKI) therapy were encouraging in patients with EGFR-mutant advanced NSCLC, though some controversy remains. The specific subgroup of patients who might benefit most from the EGFR-TKI and bevacizumab combination therapy is yet to be determined.

**Methods:** Randomized clinical trials (RCTs) that had compared the clinical efficacy of EGFR-TKI and bevacizumab combination therapy with EGFR-TKI monotherapy in treating EGFR-mutant advanced NSCLC patients published before 23 December 2022 were searched in the Cochrane, PubMed and Embase. We performed a meta-analysis for the overall survival (OS), progression-free survival (PFS), objective response rate (ORR), and treatment-related adverse events with a grade equal or more than 3 (grade≥3 TRAEs). Subgroup analyses of PFS and OS stratified by clinical characteristics and treatment were conducted.

**Results:** We included 10 RCTs involving 1520 patients. Compared with EGFR-TKI monotherapy, addition of bevacizumab to EGFR-TKI resulted in a significantly higher PFS (hazard ratio (HR) = 0.74, 95% confidence interval (95% CI): 0.62–0.87)) and ORR (risk ratio (RR) = 1.07, 95% CI: 1.01–1.13). However, no significant difference in OS (HR = 0.96, 95% CI: 0.83–1.12) was noticed. Patients with EGFR-mutant advanced NSCLC receiving combination therapy showed PFS improvement regardless of gender (male or female), Eastern Cooperative Oncology Group performance status (0 or 1), baseline central nervous system (CNS) metastasis (presence or absence) and EGFR mutation type (19del or 21L858R). Subgroup analyses showed that, with the treatment of bevacizumab and EGFR-TKI, patients who ever smoked achieved significantly better OS and PFS benefits (HR = 0.68, 95% CI: 0.48–0.95; HR = 0.59, 95% CI: 0.46–0.74, respectively), and those aged <75 years and the Asian population had significantly prolonged PFS (HR = 0.69, 95% CI: 0.52–0.91; HR = 0.71, 95% CI: 0.58–0.87; respectively). The superiority of EGFR-TKI and bevacizumab combination therapy against EGFR-TKI monotherapy in improving PFS was more significant in the erlotinib regimen subgroup. The risk of grade≥3 TRAEs was remarkably higher in the combination therapy group (HR = 1.73, 95% CI: 1.39–2.16).

**Conclusion:** Addition of bevacizumab to EGFR-TKI therapy provided significantly better PFS and ORR for EGFR-mutant advanced NSCLC patients, though with higher risk of grade≥3 TRAEs. Patients who ever smoked, aged <75 years, and Asian population might benefit more from the combination regimen.

**Systematic Review Registration:** This systematic review and meta-analysis was registered in the PROSPERO database (CRD42023401926)

## Introduction

Lung cancer is one of the most common leading causes of death worldwide (Miller and Hanna, 2021). Non-small cell lung cancer (NSCLC) and small cell lung cancer (SCLC) account for nearly 85% and 15% of all lung cancers, respectively (Molina et al., 2008; Wang et al., 2021). Epidermal growth factor receptor (EGFR), a transmembrane receptor tyrosine kinase in the ERBB family, plays fundamental role in cell proliferation and survival (Jorissen et al., 2003). The overall EGFR mutation frequency was about 50% in Asia-Pacific patients and 15%–20% in western NSCLC patients, with higher frequency in women compared with men, as well as in non-smokers compared with ever-smokers (Midha et al., 2015). Moreover, exon 19 (19del) deletion and L858R point mutation are most prevalent (Lee, 2017). The mutation and overexpression of EGFR was the pharmaceutical basis for the development and employment of EGFR-tyrosine kinase inhibitors (EGFR-TKI), and it has been widely adopted in front-line treatment for NSCLC patients with EGFR mutation (Lau et al., 2019; Li et al., 2019; Ito et al., 2020). Nevertheless, most patients inevitably develop resistance to these TKIs within 9–13 months (Lee, 2017), which has been found to be associated with increased vascular endothelial growth factor (VEGF) levels (Hung et al., 2016). It is reported that inhibition of angiogenesis could effectively enhance the anti-tumor activity of EGFR-TKI by targeting both the EGFR and VEGF pathways (Zhang et al., 2020; Watanabe et al., 2021). Therefore, addition of antiangiogenic agents might be able to prevent EGFR-TKI resistance and exert synergistic anti-tumor effects.

Bevacizumab is a kind of recombinant, anti-VEGF monoclonal antibody, which targets vascular endothelial growth factor-A (Goyal et al., 2022). The addition of bevacizumab to chemotherapy or immune check point inhibitors in the treatment of advanced NSCLC was demonstrated to be favorable (Systematic review and meta, 2013; Socinski et al., 2021; Sugawara et al., 2021), whereas its role in EGFR-TKI combination therapy remains controversy. The combination of erlotinib and bevacizumab was shown to be encouraging and has been accepted as an alternative choice of front-line therapy (Hsu et al., 2018). However, in the trials that mostly included non-Asian patients, no superiority of the combination regimen was found in terms of the anti-tumor effect, as compared with EGFR-TKI alone (Stinchcombe et al., 2019; Soo et al., 2021).

As more clinical trials had reported the outcome of combination therapy involving bevacizumab and different EGFR-TKIs, the present study aimed to clarify the clinical value of bevacizumab and EGFR-TKI combination therapy in EGFR-mutant advanced NSCLC patients, and further explore its role in predefined subgroups, in an attempt to provide evidence for selection of NSCLC individuals who might benefit most by adding bevacizumab to EGFR-TKI.

## Methods

### Search strategy

This systematic review and meta-analysis was registered in the International prospective register of systematic reviews (PROSPERO) database (CRD42023401926). We conducted a thorough search to identify relevant RCTs that had compared the clinical efficacy of combination EGFR-TKI and bevacizumab therapy with EGFR-TKI monotherapy in the treatment of advanced NSCLC using the following databases: PUBMED, EMBASE, and Cochrane. The last retrieval was performed on 23 December 2022. The keywords used were as follows: all terms related to “NSCLC,” “bevacizumab,” “erlotinib,” “gefitinib,” “icotinib,” “afatinib,” “Osimertinib,” and other EGFR-TKIs, “epidermal growth factor receptor,” “EGFR,” all terms related to clinical trial. The retrieval strategy for the PubMed database is listed in Supplementary Table S1.

### Eligibility criteria

Studies fulfilling all the following criteria were included (Miller and Hanna, 2021) RCTs; (Molina et al., 2008) studies that had compared combination EGFR-TKI and bevacizumab therapy with EGFR-TKI monotherapy in treating advanced NSCLC (Wang et al., 2021); studies included patients with EGFR mutations (Jorissen et al., 2003); with at least one of the following reported outcomes: overall survival (OS), progression-free survival (PFS), objective response rate (ORR) and treatment-related adverse events with a grade equal or more than 3 (grade≥3 TRAEs) (Midha et al., 2015); studies with a sample size of at least 40 patients. For the overlapping reports obtained from the same group of patients, the latest and most complete reports were included. Duplicate publications, review articles, meta-analyses, editorials, case reports, letters, animal or cellular experiments and studies with incomplete data were excluded.

### Data extraction

Data extraction was performed independently by two investigators according to the predefined criteria. The information extracted from each study was as follows: the name of study, year of publication, trial number and design, ethnicity involved, sample size (female%), treatment regimens, follow-up time, age (median, range, years), Eastern Cooperative Oncology Group performance status (ECOG PS), smoking status, baseline central nervous system (CNS) metastasis condition, pathological features, EGFR mutation status, outcomes including PFS, OS, ORR and grade≥3 TRAEs. A third investigator was consulted when there were any disagreements during the process, and the discrepancies were resolved by discussion.

### Quality assessment

The quality assessment of included trials was conducted independently by two investigators. The quality of RCT was evaluated according to the Cochrane Collaboration tool, with a total of 6 items included: selection bias, performance bias, detection bias, attrition bias, reporting bias, and other bias (Supplementary Figure S1). There are three levels for each item, that is, a high, low or unclear risk of bias. A third investigator was consulted when there were any disagreements during the process, and the discrepancies were resolved by discussion.

### Statistical analysis

R software (version 4.1.0) with package meta was adopted to perform meta-analysis. The primary outcomes were OS and PFS, and the secondary outcomes were ORR and grade≥3 TRAEs. Hazard ratios (HR) with 95% CIs for OS and PFS, odds ratios (OR) with 95% CIs for ORR and grade≥3 TRAEs were extracted from the original report.

For each outcome, statistical heterogeneity was evaluated using the Cochran’s Q test and the *I* ^*2*^ measure. An *I* ^*2*^ value greater than 50% or *p*-value equal or less than 0.1 is generally considered to indicate a substantial level of heterogeneity, which requires a random effects model for pooled analysis and initiates subsequent sensitivity analysis to identify the source. Otherwise, a fixed effects model was adopted. The Egger regression test with a funnel plot was used to evaluate the publication bias, and a *p*-value of less than 0.10 was considered to indicate significant asymmetry and publication bias. When there was publication bias, trim-and-fill method was used for data correction. Subgroup analyses were conducted with the following stratifications: gender, age, baseline CNS metastasis, EGFR mutation type, smoking status, different type of EGFR-TKI, treatment line, ethnicity, and ECOG PS.

## Results

### Study selection and characteristics

**FIGURE 1:**
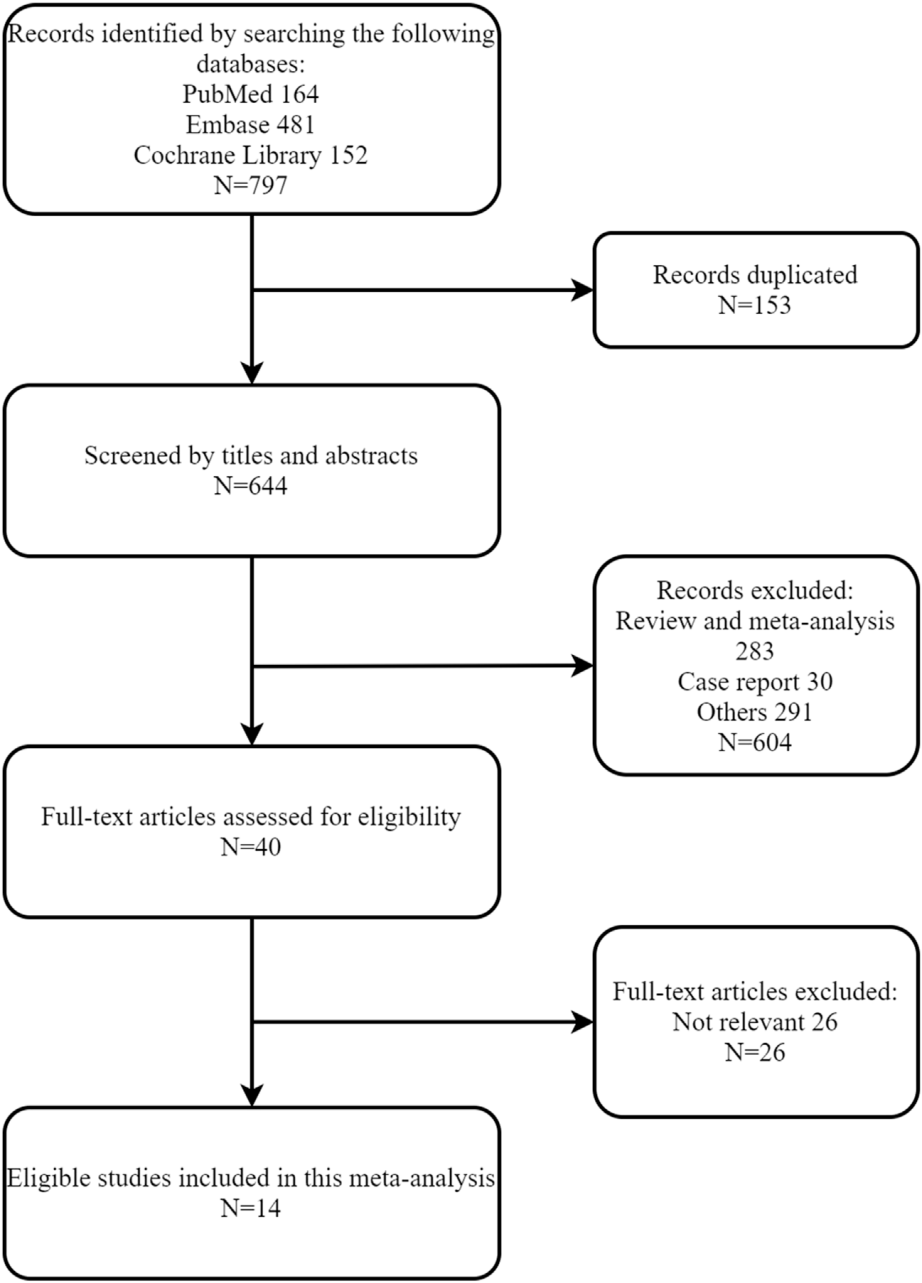
Flowchart of study selection.

We identified 797 records from the databases. After excluding 153 duplicates and 604 reports for irrelevant titles and abstracts, a total of 40 studies were reviewed for full-text assessment. Finally, 14 studies from 10 trials were included in our work (Seto et al., 2014; Kato et al., 2018; Saito et al., 2019; Stinchcombe et al., 2019; Akamatsu et al., 2021; Soo et al., 2021; Yamamoto et al., 2021; Zhou et al., 2021; Ishikawa et al., 2022; Kawashima et al., 2022; Kenmotsu et al., 2022; Lee et al., 2022; Nakamura et al., 2022; Piccirillo et al., 2022), with 1 trial only reported in conference abstract (Ishikawa et al., 2022) (Figure 1).

The detailed information of the 14 studies were shown in Table 1 and Table 2. A total of 1520 patients were included in our work, with 760 in the combination therapy group and 760 in the monotherapy group. One out of 10 trials had evaluated the efficacy of afatinib plus bevacizumab as compared with afatinib alone (Ishikawa et al., 2022), 6 had compared erlotinib plus bevacizumab with erlotinib alone (Seto et al., 2014; Kato et al., 2018; Saito et al., 2019; Stinchcombe et al., 2019; Yamamoto et al., 2021; Zhou et al., 2021; Kawashima et al., 2022; Lee et al., 2022; Piccirillo et al., 2022), and 3 had compared osimertinib plus bevacizumab with osimertinib monotherapy (Akamatsu et al., 2021; Kenmotsu et al., 2022; Nakamura et al., 2022). There were 3 phase III RCTs and 7 phase II RCTs. The majority of the included patient population was Asian. There were 8 RCTs adopted the EGFR-TKI regimen as first-line treatment (Seto et al., 2014; Saito et al., 2019; Stinchcombe et al., 2019; Zhou et al., 2021; Ishikawa et al., 2022; Kenmotsu et al., 2022; Lee et al., 2022; Piccirillo et al., 2022). Most patients were ECOG PS 0-1.

**TABLE 1:** Baseline characteristics of included studies in the meta-analysis.

Study/year	Design	Histology/Stage	Treatment		Treatment line	Median follow-up	Randomization	Outcomes
AfaBev-CS (2022)(26) jRCTs061180006	Phase II	Non-squamous NSCLC	Afatinib (30 mg) once daily + bevacizumab (15 mg/kg) every 21 days	Afatinib once daily (40 mg)	First-line	31.3 months	1:1	PFS, ORR, AEs
ARTEMIS-CTONG1509 (2021)(25) NCT02759614	Phase III	NSCLC/Stage IIIB-IV, recurrence	Erlotinib (150 mg) once daily + bevacizumab (15 mg/kg) every 21 days	Erlotinib once daily (150 mg)	First-line	NA	1:1	PFS, OS, ORR, AEs
BEVERLY (2022)(31) NCT02633189	Phase III	NSCLC/Stage IIIB, IV	Erlotinib (150 mg) once daily + bevacizumab (15 mg/kg) every 21 days	Erlotinib once daily (150 mg)	First-line	36.3 months	1:1	PFS, OS, ORR, AEs
BOOSTER (2021)(18) NCT03133546	Phase II	Non-squamous NSCLC/Stage IIIB-IV	Osimertinib (80 mg) once daily + bevacizumab (15 mg/kg) every 21 days	Osimertinib once daily (80 mg)	Second-line	33.8 months	1:1	PFS, OS, ORR, AEs
JO25567 (2014)(20) JapicCTI-111390	Phase II	Non-squamous NSCLC/Stage IIIB-IV, recurrence	Erlotinib (150 mg) once daily + bevacizumab (15 mg/kg) every 21 days	Erlotinib once daily (150 mg)	First-line	20.4 months for PFS	1:1	PFS, ORR
JO25567 (2018)(24) JapicCTI-111390	Phase II	Non-squamous NSCLC/Stage IIIB-IV, recurrence	Erlotinib (150 mg) once daily + bevacizumab (15 mg/kg) every 21 days	Erlotinib once daily (150 mg)	First-line	34.7 months for OS	1:1	OS
JO25567 (2018)(21) JapicCTI-111390	Phase II	Non-squamous NSCLC/Stage IIIB-IV, recurrence	Erlotinib (150 mg) once daily + bevacizumab (15 mg/kg) every 21 days	Erlotinib once daily (150 mg)	First-line	27 months for monotherapy; 25.9 months for combination therapy	1:1	AEs
NEJ026 (2019)(22) UMIN000017069	Phase III	Non-squamous NSCLC/Stage IIIB–IV	Erlotinib (150 mg) once daily + bevacizumab (15 mg/kg) every 21 days	Erlotinib once daily (150 mg)	First-line	12.4 months for PFS	1:1	PFS, ORR, AEs
NEJ026 (2022)(27) UMIN000017069	Phase III	Non-squamous NSCLC/Stage IIIB–IV	Erlotinib (150 mg) once daily + bevacizumab (15 mg/kg) every 21 days	Erlotinib once daily (150 mg)	First-line	39.2 months for OS	1:1	OS
Stinchcombe et al. (2019)(19) NCT01532089	Phase II	Non-squamous NSCLC	Erlotinib (150 mg) once daily + bevacizumab (15 mg/kg) every 21 days	Erlotinib once daily (150 mg)	First-line	33 months	1:1	PFS, OS, ORR
WJOG8715L (2021)(23) UMIN000023761	Phase II	NSCLC/Stage IIIB-IV, recurrence	Osimertinib (80 mg) once daily + bevacizumab (15 mg/kg) every 21 days	Osimertinib once daily (80 mg)	Not First-line	16.2 months for monotherapy; 16.0 months for combination therapy	1:1	PFS, OS, ORR, AEs
WJOG9717L (2022)(28) UMIN000030206	Phase II	Non-squamous NSCLC/Stage IIIB-IV, recurrence	Osimertinib (80 mg) once daily + bevacizumab (15 mg/kg) every 21 days	Osimertinib once daily (80 mg)	First-line	19.8 months	1:1	ORR, AEs
WJOG9717L (2022)(30) UMIN000030206	Phase II	Non-squamous NSCLC/Stage IIIB-IV, recurrence	Osimertinib (80 mg) once daily + bevacizumab (15 mg/kg) every 21 days	Osimertinib once daily (80 mg)	First-line	36 months	1:1	Updated PFS, OS
Youngjoo Lee et al. (2022)(29) NCT03126799	Phase II	Stage IIIB/IV NSCLC	Erlotinib (150 mg) once daily + bevacizumab (15 mg/kg) every 21 days	Erlotinib once daily (150 mg)	First-line	38.9 months	1:1	PFS, OS, ORR, AEs

**TABLE 2:** Baseline characteristics of patients included in the meta-analysis.

Study/year	Treatment	Patients (n, female%)	Age (median, years)	Smoking history (never smoker, smoker, other)	Ethnicity	EGFR mutation (19del, L858R, other)	ECOG score (0, 1, 2)	CNS metastasis
AfaBev-CS (2022)(26) jRCTs061180006	Afatinib + Bev	50	NA	NA	Japanese centers	NA	NA	NA
	Afatinib	50	NA	NA	Japanese centers	NA	NA	NA
ARTEMIS-CTONG1509 (2021)(25) NCT02759614	Erlotinib + Bev	157 (61.8)	57 (33–78)	NA	Asian (100%)	(82, 75, 0)	(25, 132, 0)	44 (28%)
	Erlotinib	154 (62.3)	59 (27–77)	NA	Asian (100%)	(79, 75, 0)	(17, 137, 0)	47 (30.5%)
BEVERLY (2022)(31) NCT02633189	Erlotinib + Bev	80 (65%)	65.9 (57.9–71.8)	(46, 34, 0)	Italian centers	(44, 34, 2)	(52, 26, 2)	None
	Erlotinib	80 (62.5%)	67.7 (60.7–73.6)	(37, 43, 0)	Italian centers	(44, 32, 4)	(47, 29, 4)	None
BOOSTER (2021)(18) NCT03133546	Osimertinib + Bev	78 (60.3%)	68 (34–85)	(44, 34, 0)	Asian (41%)	(58, 20, 0)	(22, 51, 5)	13 (16.7%)
	Osimertinib	77 (63.6%)	66 (41–83)	(49, 28, 0)	Asian (40.3%)	(51, 26, 0)	(25, 48, 4)	8 (10.4%)
JO25567 (2014)(20) JapicCTI-111390	Erlotinib + Bev	75 (60%)	67 (59–73)	(42, 9, 24)^*^	Asian (100%)	(40, 35, 0)	(43, 32, 0)	None
	Erlotinib	77 (66%)	67 (69–73)	(45, 6, 26)^*^	Asian (100%)	(40, 37, 0)	(41, 36, 0)	None
NEJ026 (2019)(22) UMIN000017069	Erlotinib + Bev	112 (63%)	67 (61–73)	(65, 47, 0)	Asian (100%)	(56, 56, 0)	(64, 48, 0)	36 (32%)
	Erlotinib	112 (65%)	68 (62–73)	(64, 48, 0)	Asian (100%)	(55, 57, 0)	(68, 42, 2)	36 (32%)
Stinchcombe et al. (2019)(19) NCT01532089	Erlotinib + Bev	43 (72%)	65 (31–84)	(25, 17, 1)	Non-Asian (96%)	(29, 14, 0)	(24, 19, 0)	11 (26%)
	Erlotinib	45 (69%)	63 (47–84)	(23, 22, 0)	Non-Asian (94%)	(30, 15, 0)	(19, 26, 0)	14 (31%)
WJOG8715L (2021)(23) UMIN000023761	Osimertinib + Bev	40 (60%)	68 (43–82)	(21, 19, 0)	Asian (100%)	(22, 18, 0)	(20, 20, 0)	12 (30%)
	Osimertinib	41 (59%)	70 (41–82)	(20, 21, 0)	Asian (100%)	(28, 13, 0)	(17, 24, 0)	9 (22%)
WJOG9717L (2022)(28) UMIN000030206	Osimertinib + Bev	61 (60.7%)	67 (59–74)	(38, 23, 0)	Asian (100%)	(35, 26, 0)	(32, 29, 0)	NA
	Osimertinib	61 (62.3%)	66 (60–74)	(30, 31, 0)	Asian (100%)	(36, 25, 0)	(34, 27, 0)	NA
Youngjoo Lee et al. (2022)(29) NCT03126799	Erlotinib + Bev	64 (68.8%)	31 (48.4%)^#^	(41, 23, 0)	Asian (100%)	(37, 27, 0)	(33, 31, 0)	29 (45.3%)
	Erlotinib	63 (63.5%)	24 (38.1%)^#^	(42, 21, 0)	Asian (100%)	(37. 26, 0)	(28, 35, 0)	30 (47.6%)

*indicates (never smoker, former light smoker, other).

#indicates number of participants who aged ≥65 years (percentage%).

### Overall population

**FIGURE 2:**
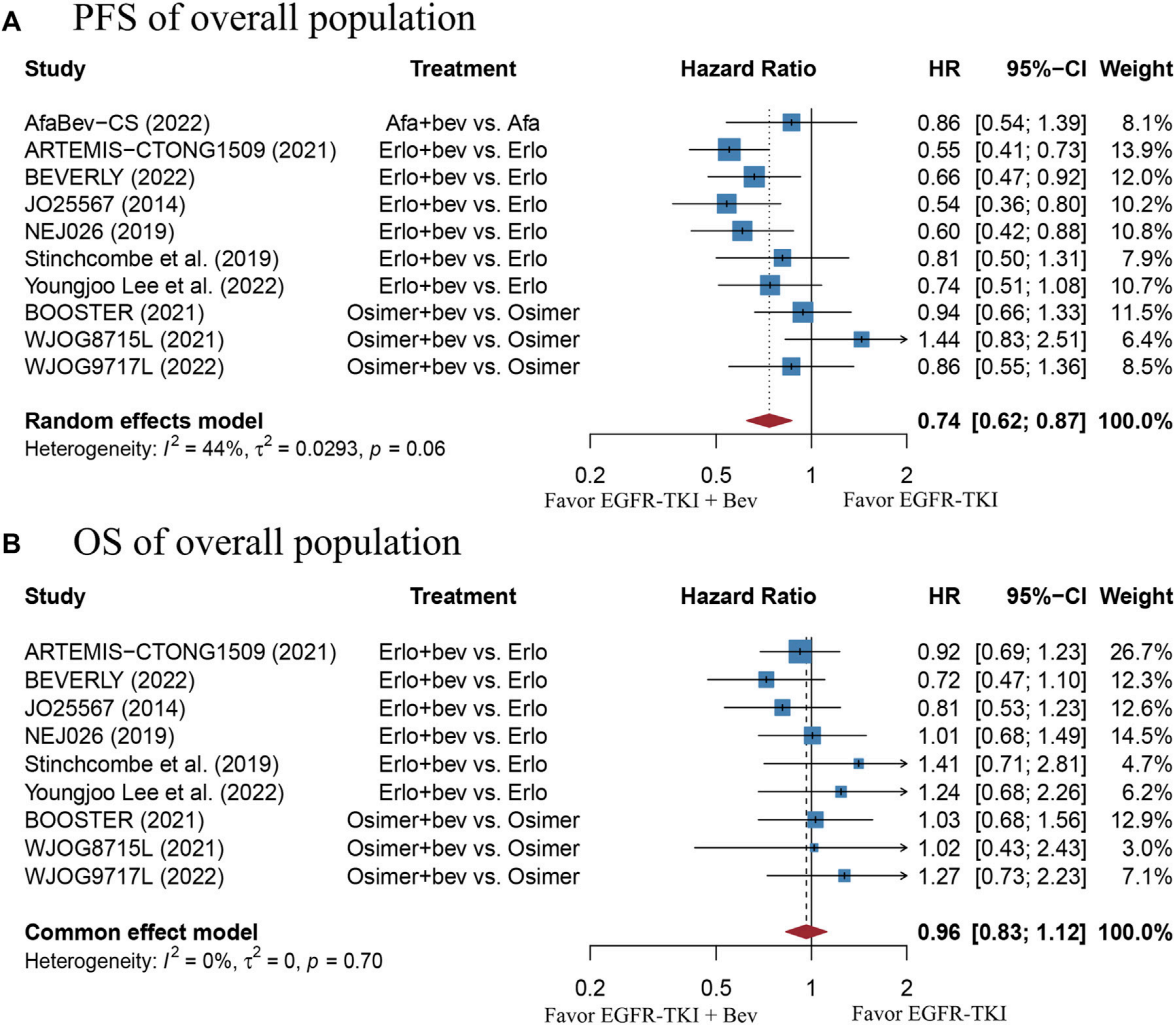
**(A)** Forest plot of HRs for PFS in the overall population. **(B)** Forest plot of HRs for OS in the overall population. Afa, Afatinib; Bev, Bevacizumab; Erlo, Erlotinib; Osimer, Osimertinib; CI, confidence interval.

There were 10 studies involving 1520 patients with EGFR-mutant advanced NSCLC eligible for the pooling analysis of PFS. The pooled PFS result derived from a random-effect model showed that the combination therapy group had a significantly longer PFS as compared with the EGFR-TKI monotherapy group (HR = 0.74, 95% CI: 0.62–0.87, Cochran’s Q *p* = 0.06, *I* ^2^ = 44%; Figure 2A). The funnel plot and Egger’s test both demonstrated publication bias (Supplementary Figure S2A, *p* = 0.0227). Thus, trim-and-fill method was adopted. The data after correction also suggested significant PFS benefit in the combination therapy group (HR = 0.655, 95% CI: 0.5439–0.7889; Supplementary Figure S2B). Sensitivity analysis showed that removal of any study did not affect the pooled HR, which indicates stability of the result (Supplementary Figure S3A).

A total of 9 studies including 1420 patients with EGFR-mutant advanced NSCLC were enrolled for the pooling analysis of OS. The pooled HR was 0.96 (95% CI: 0.83–1.12), with no heterogeneity (Cochran’s Q *p* = 0.7, *I* ^2^ = 0%; Figure 2B), suggesting that there was no significant difference in OS between the combination therapy group and EGFR-TKI monotherapy group. The funnel plot and Egger’s test showed no publication bias (Supplementary Figure S2C, *p* = 0.1486). Sensitivity analysis showed that removal of any study did not affect the pooled HR, which indicates stability of the result (Supplementary Figure S3B).

**FIGURE 3:**
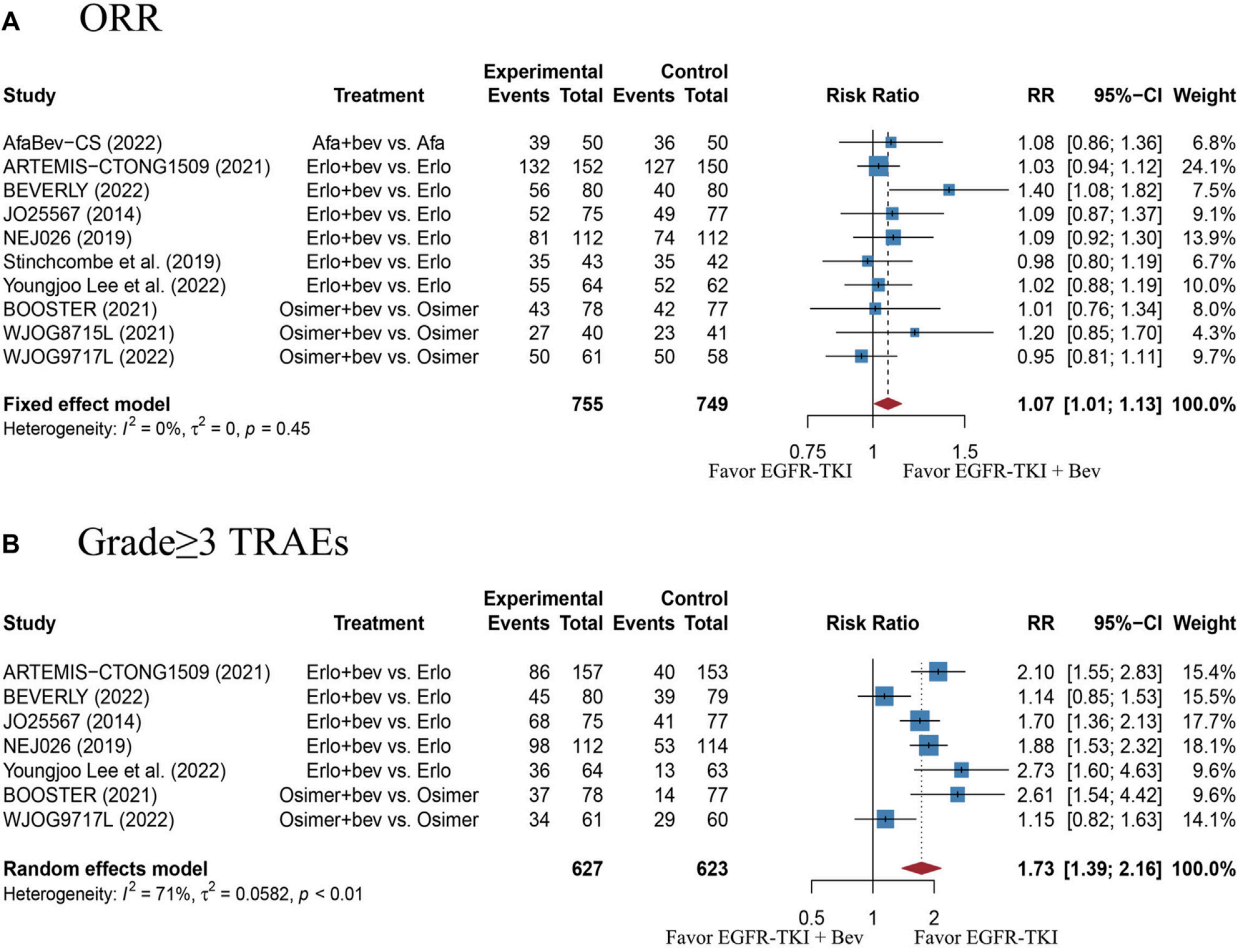
**(A)** Forest plot of RRs for ORR in the overall population. **(B)** Forest plot of RRs for grade≥3 TRAEs in the overall population. Afa, Afatinib; Bev, Bevacizumab; Erlo, Erlotinib; Osimer, Osimertinib; CI, confidence interval.

There were 10 studies with 1520 EGFR-mutant advanced NSCLC patients provided the ORR outcome. The pooled RR was 1.07 (95% CI: 1.01–1.13), with no heterogeneity (Cochran’s Q *p* = 0.45, *I* ^2^ = 0%; Figure 3A), indicating a slightly better response in the combination therapy group, as compared with the EGFR-TKI monotherapy group. The funnel plot and Egger’s test showed no publication bias (Supplementary Figure S2D, *p* = 0.1524). Nevertheless, sensitivity analysis showed that removal of the BEVERLY research would affect the pooled RR, which indicates instability of the result (Supplementary Figure S3C). This data should be interpreted with caution.

There were 7 studies with 1250 EGFR-mutant advanced NSCLC patients reported data on grade≥3 TRAEs. The pooled RR was 1.73 (95% CI: 1.39–2.16), with high heterogeneity (Cochran’s Q *p* < 0.01, *I* ^2^ = 71%; Figure 3B), which suggests a significantly higher risk of grade≥3 TRAEs with combination therapy, as compared with the EGFR-TKI monotherapy. The funnel plot and Egger’s test showed no publication bias (Supplementary Figure S2E, *p* = 0.6441). Sensitivity analysis showed that removal of any study did not affect the pooled RR, indicating stability of the result (Supplementary Figure S3D). Moreover, the most reported grade≥3 TRAEs were listed in Supplementary Table S2. Of those, the increased risks of hypertension, proteinuria and rash in the combination therapy group were statistically significant, as compared with monotherapy group.

### Subgroup analyses

Subgroup analyses of PFS and OS were conducted with the following stratifications: gender, age, baseline CNS metastasis condition, EGFR mutation type, smoking status, different type of EGFR-TKI, treatment line, ethnicity, and ECOG PS (Table 3).

**TABLE 3:** Subgroup analyses of progression-free survival and overall survival.

Subgroup	Studies (patients, n)	HR for PFS (95%CI)	Heterogeneity *p*-value, *I* ^2^ (%)	Studies (patients, n)	HR for OS (95%CI)	Heterogeneity *p*-value, *I* ^2^ (%)
Gender						
Male	8 (494)	0.63 (0.51–0.78)	*p* = 0.2, *I* ^2^ = 29%	4 (253)	0.92 (0.65–1.3)	*p* = 0.29, *I* ^2^ = 21%
Female	8 (838)	0.76 (0.59–0.97)	*p* = 0.06, *I* ^2^ = 48%	4 (438)	0.86 (0.66–1.12)	*p* = 0.44, *I* ^2^ = 0%
Age (years)						
<75	5 (770)	0.69 (0.52–0.91)	*p* = 0.09, *I* ^2^ = 50%	NA	NA	NA
≥75	4 (114)	0.6 (0.33–1.09)	*p* = 0.26, *I* ^2^ = 26%	NA	NA	NA
ECOG PS						
0	8 (568)	0.68 (0.55–0.84)	*p* = 0.15, *I* ^2^ = 35%	4 (362)	0.86 (0.63–1.18)	*p* = 0.92, *I* ^2^ = 0%
1	8 (756)	0.71 (0.59–0.84)	*p* = 0.49, *I* ^2^ = 0%	4 (321)	0.87 (0.66–1.16)	*p* = 0.39, *I* ^2^ = 0%
Baseline CNS metastasis						
Yes	5 (284)	0.63 (0.47–0.85)	*p* = 0.58, *I* ^2^ = 0%	NA	NA	NA
No	7 (873)	0.70 (0.56–0.88)	*p* = 0.09, *I* ^2^ = 45%	NA	NA	NA
Smoking status						
Never-smoker	7 (599)	0.9 (0.66–1.24)	*p* = 0.03, *I* ^2^ = 58%	4 (407)	1.05 (0.8–1.38)	*p* = 0.39, *I* ^2^ = 0%
Smoker	7 (409)	0.59 (0.46–0.74)	*p* = 0.43, *I* ^2^ = 0%	4 (271)	0.68 (0.48–0.95)	*p* = 0.15, *I* ^2^ = 43%
EGFR mutation type						
19del	7 (694)	0.68 (0.57–0.82)	*p* = 0.35, *I* ^2^ = 11%	5 (549)	1.03 (0.78–1.35)	*p* = 0.77, *I* ^2^ = 0%
L858R	7 (551)	0.67 (0.54–0.83)	*p* = 0.43, *I* ^2^ = 0%	5 (447)	0.85 (0.63–1.14)	*p* = 0.63, *I* ^2^ = 0%
Ethnicity						
Asian	8 (1180)	0.71 (0.58–0.87)	*p* = 0.07, *I* ^2^ = 46%	7 (1080)	0.96 (0.81–1.15)	*p* = 0.84, *I* ^2^ = 0%
Non-Asian	3 (340)	0.84 (0.59–1.19)	*p* = 0.12, *I* ^2^ = 52%	3 (340)	1.03 (0.67–1.58)	*p* = 0.14, *I* ^2^ = 50%
Different type of EGFR-TKI						
Afatinib	1 (100)	0.86 (0.54–1.39)	NA	NA	NA	NA
Erlotinib	6 (1062)	0.63 (0.54–0.73)	*p* = 0.65, *I* ^2^ = 0%	6 (1062)	0.93 (0.78–1.1)	*p* = 0.51, *I* ^2^ = 0%
Osimertinib	3 (358)	1 (0.78–1.28)	*p* = 0.34, *I* ^2^ = 8%	3 (358)	1.1 (0.8–1.5)	*p* = 0.83, *I* ^2^ = 0%
Treatment line						
First-line	8 (1284)	0.66 (0.58–0.76)	*p* = 0.49, *I* ^2^ = 0%	7 (1184)	0.95 (0.81–1.12)	*p* = 0.5, *I* ^2^ = 0%
Non-first-line	2 (291)	1.06 (0.79–1.43)	*p* = 0.2, *I* ^2^ = 38%	2 (291)	1.03 (0.71–1.5)	*p* = 0.98, *I* ^2^ = 0%

**FIGURE 4:**
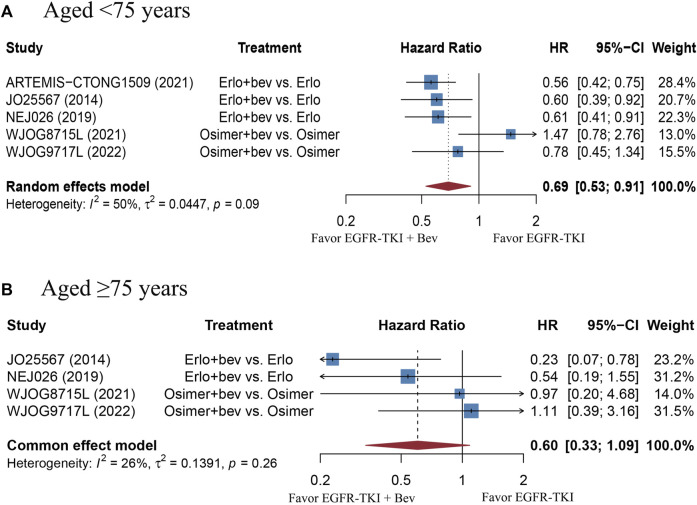
**(A)** Forest plot of HRs for PFS in patients aged less than 75 years old. **(B)** Forest plot of HRs for OS in patients aged equal or more than 75 years old. Afa, Afatinib; Bev, Bevacizumab; Erlo, Erlotinib; Osimer, Osimertinib; CI, confidence interval.

**FIGURE 5:**
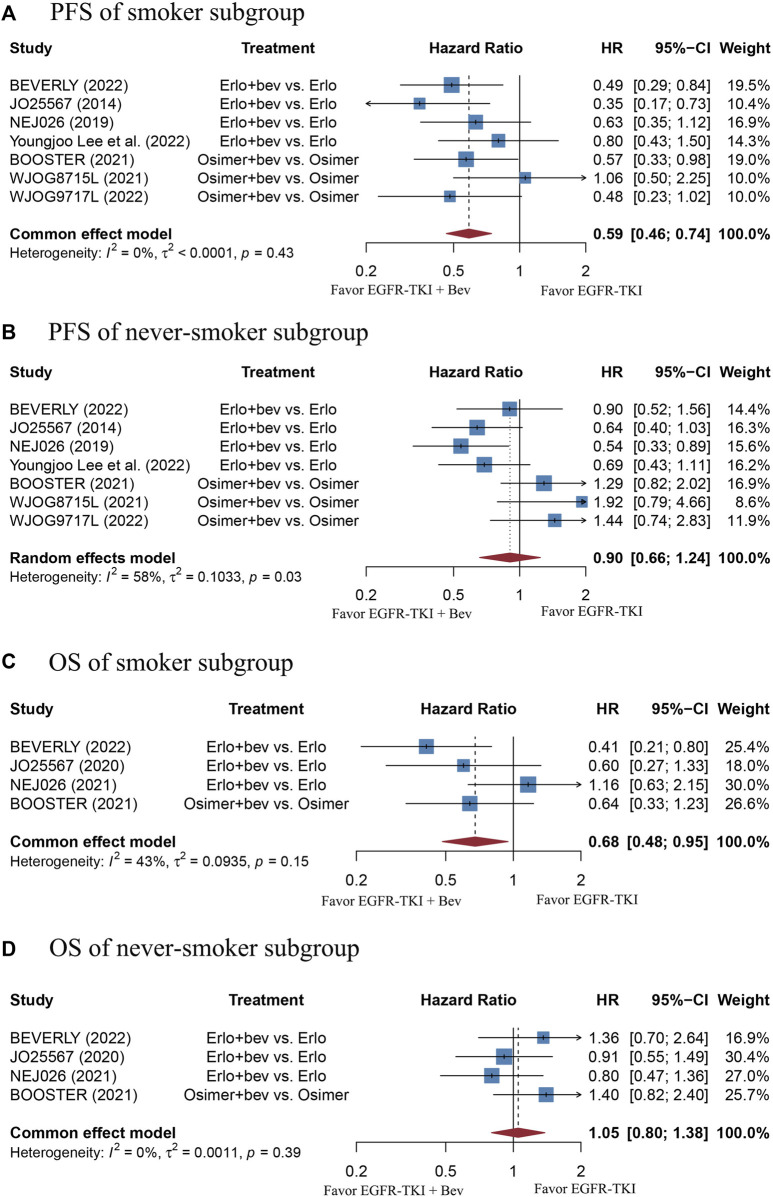
**(A)** Forest plot of HRs for PFS in smoker subgroup. **(B)** Forest plot of HRs for PFS in never-smoker subgroup. **(C)** Forest plot of HRs for OS in smoker subgroup. **(D)** Forest plot of HRs for OS in never-smoker subgroup. Note: There were 13 former light smokers from the NEJ026 study excluded from the analysis. The 15 former light smokers from the JO25567 study were included in the never-smoker subgroup. Afa, Afatinib; Bev, Bevacizumab; Erlo, Erlotinib; Osimer, Osimertinib; CI, confidence interval.

**FIGURE 6:**
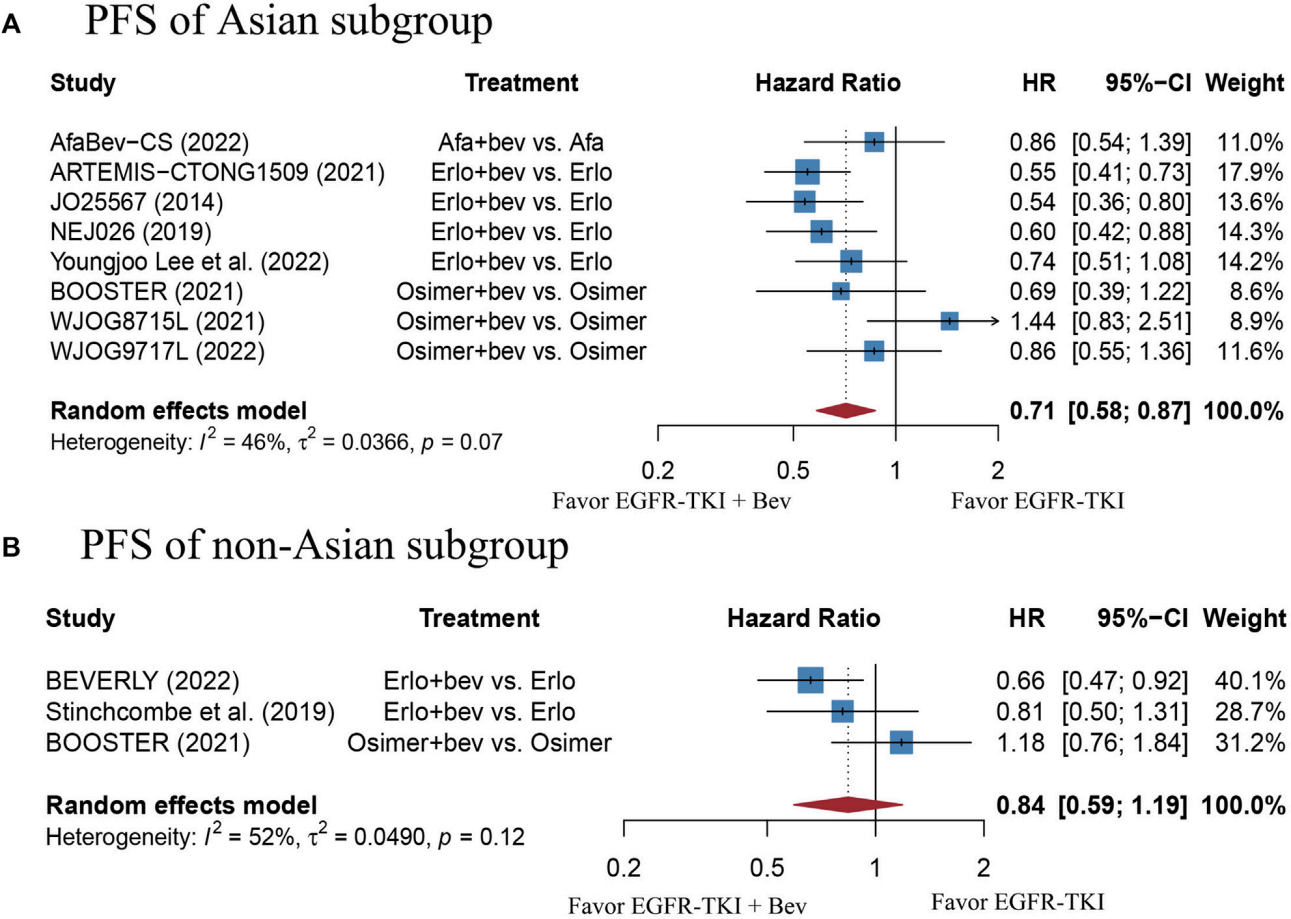
**(A)** Forest plot of HRs for PFS in Asian subgroup. **(B)** Forest plot of HRs for PFS in non-Asian subgroup. Note: The BEVERLY study conducted in Italian centers and the work proposed by Stinchcombe et al. including mostly non-Asian people were both categorized as non-Asian group. Afa, Afatinib; Bev, Bevacizumab; Erlo, Erlotinib; Osimer, Osimertinib; CI, confidence interval.

**FIGURE 7:**
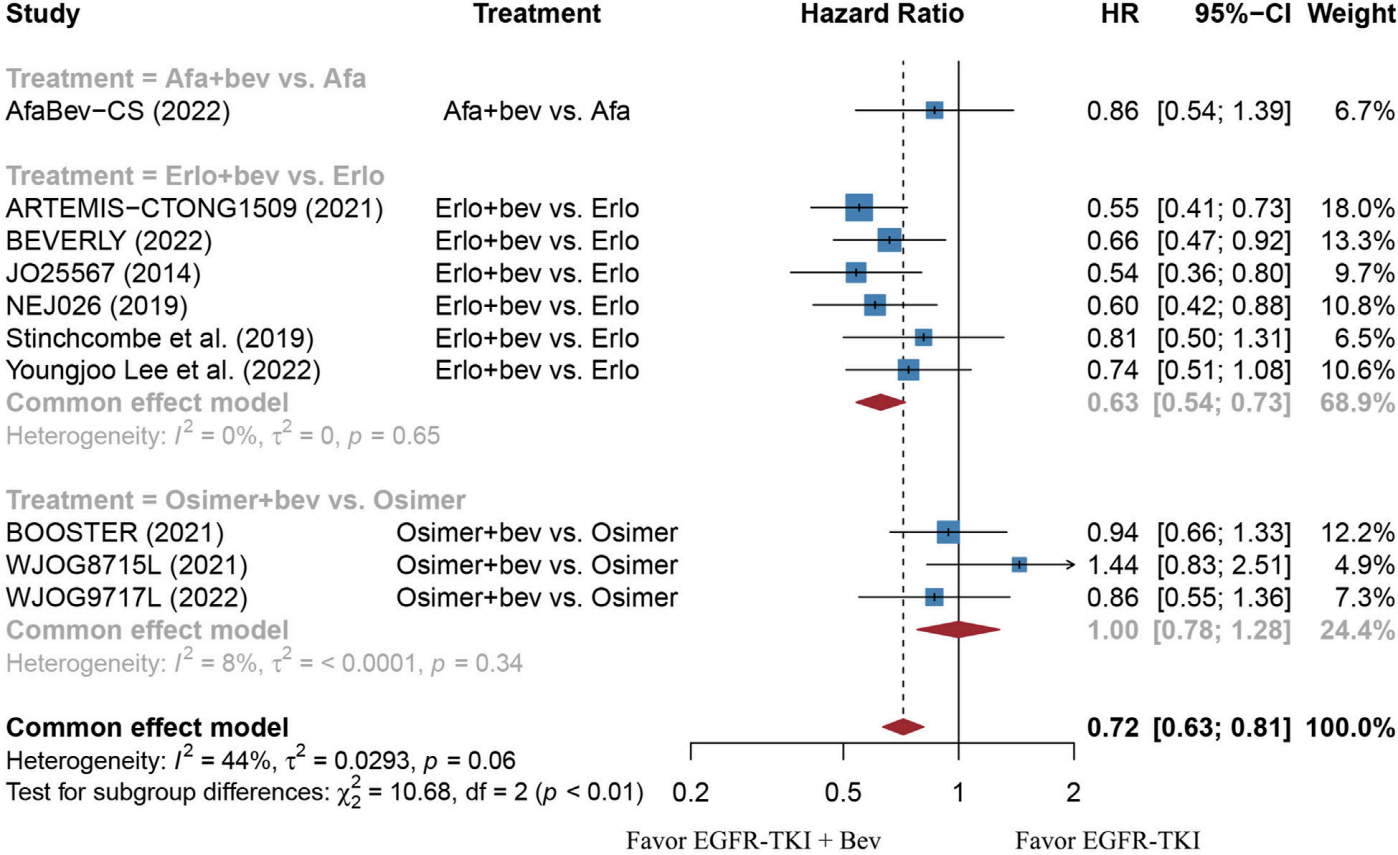
Forest plot of HRs for PFS based on different types of EGFR-TKI. Afa, Afatinib; Bev, Bevacizumab; Erlo, Erlotinib; Osimer, Osimertinib; CI, confidence interval.

**FIGURE 8:**
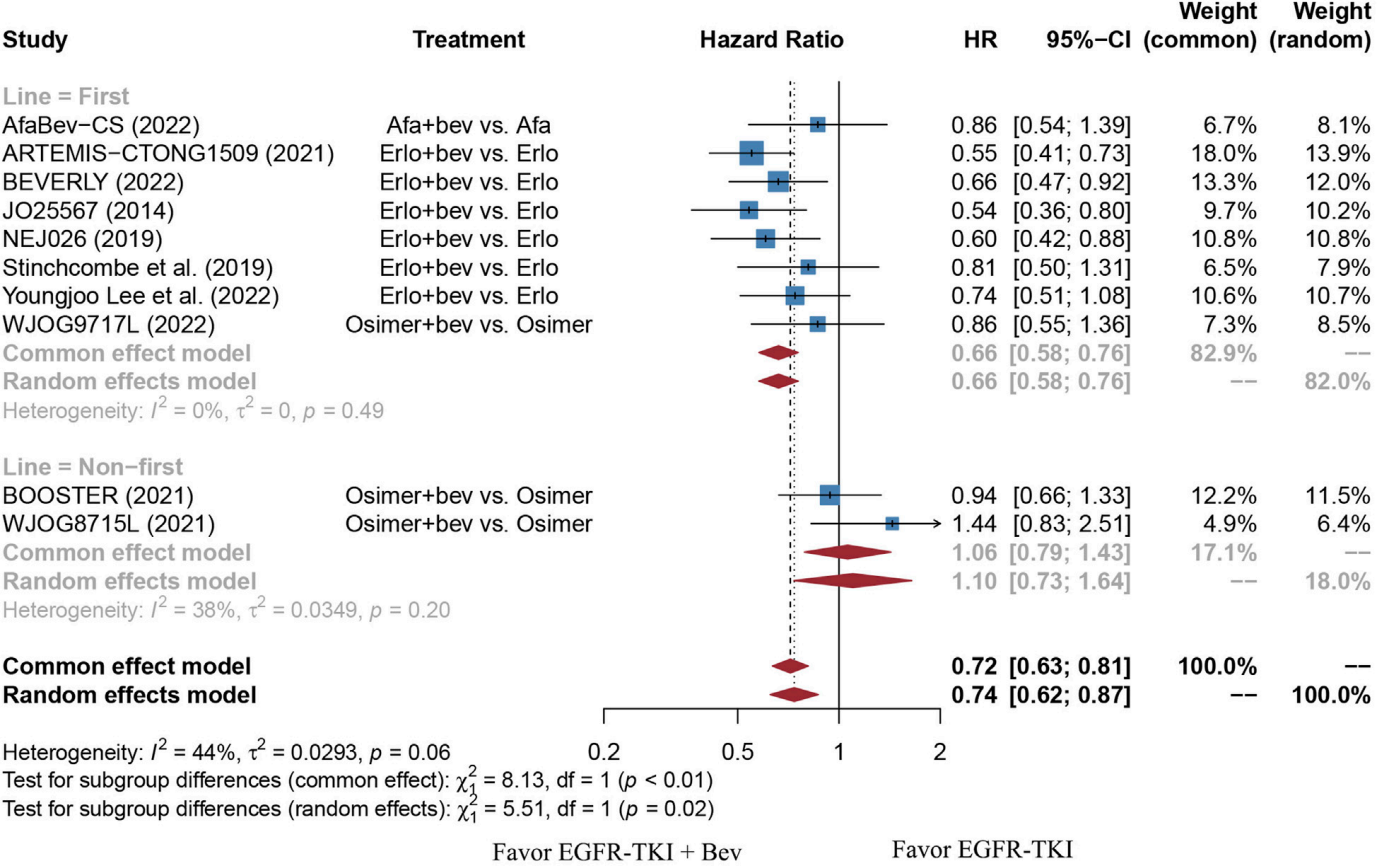
Forest plot of HRs for PFS based on different treatment lines. Afa, Afatinib; Bev, Bevacizumab; Erlo, Erlotinib; Osimer, Osimertinib; CI, confidence interval.

The stratified analysis showed that addition of bevacizumab to EGFR-TKI therapy could significantly improve the PFS for all EGFR-mutant advanced NSCLC patients irrespective of the differences in gender, EGFR mutation type, ECOG PS, and baseline CNS metastasis (Table 3). However, significant PFS benefit of combination therapy was noticed in patients with age below 75 years (HR = 0.69, 95% CI: 0.52–0.91, Cochran’s Q *p* = 0.09, *I* ^2^ = 50%; Figures 4A,B), in the smoker population (HR = 0.59, 95% CI: 0.46–0.74, Cochran’s Q *p* = 0.43, *I* ^2^ = 0%; Figures 5A,B), and in the Asian population (HR = 0.71, 95% CI: 0.58–0.87, Cochran’s Q *p* = 0.07, *I* ^2^ = 46%; Figures 6A,B). Moreover, patients treated with erlotinib and bevacizumab combination therapy yielded remarkably better PFS (HR = 0.63, 95% CI: 0.54–0.73, Cochran’s Q *p* = 0.65, *I* ^2^ = 0%; Figure 7), whereas those treated with osimertinib or afatinib and bevacizumab had comparable efficacy with those treated with EGFR-TKI monotherapy (For osimertinib, HR = 1, 95% CI: 0.78–1.28, Cochran’s Q *p* = 0.34, *I* ^2^ = 8%; Figure 7). Further analyses revealed that EGFR-TKI and bevacizumab had significantly better PFS outcome when adopted as first-line treatment (HR = 0.66, 95% CI: 0.58–0.76, Cochran’s Q *p* = 0.49, *I* ^2^ = 0%; Figure 8).

Adding bevacizumab to EGFR-TKI therapy did not affect the OS for all EGFR-mutant advanced NSCLC patients, regardless of their gender, EGFR mutation type, different type of EGFR-TKI, treatment line, and ECOG PS (Table 3). Interestingly, significant OS benefit of combination therapy was observed in the smoker subgroup, with no heterogeneity (HR = 0.68, 95%CI: 0.48–0.95, Cochran’s Q *p* = 0.15, *I* ^2^ = 43%; Figures 5C,D).

## Discussion

The results of this meta-analysis showed that adding bevacizumab to EGFR-TKI therapy provided significantly better PFS and ORR results for NSCLC patients harboring EGFR mutations, though this benefit failed to translate into prolonging OS. The subgroup analyses stratified by patients’ clinical features also proved that EGFR-TKI and bevacizumab combination therapy consistently resulted in longer PFS regardless of the gender, ECOG PS, baseline CNS metastasis and EGFR mutation type. Interestingly, in the smoker subgroup (former or current smoker), addition of bevacizumab to EGFR-TKI could significantly prolong the PFS and OS. Moreover, as compared with those aged equal or more than 75 years, combination therapy provided with significantly favorable PFS results for EGFR-mutant advanced NSCLC patients who aged less than 75 years.

VEGF, a family of polypeptide growth factors, mainly included VEGF-A, -B, -C and -D (32). Of those, VEGF-A is the most investigated variant, which primarily binds to VEGF receptor 1 and 2, thus inducing angiogenesis (Ferrara and Adamis, 2016). Bevacizumab, a humanized monoclonal antibody directed against VEGF-A, has been approved for the treatment of NSCLC globally. Given that the VEGF and EGFR pathways share common downstream signaling pathway that regulate cellular proliferation, it is suggested that EGFR-mutant tumors are more VEGF-dependent, thus dual inhibition of EGFR and VEGF might yield better antitumor effects (Abid et al., 2004; Le et al., 2021). In addition, it has been found that VEGF contributes to the acquired EGFR-TKI resistance, which supports the hypothesis that dual inhibition of EGFR and VEGF could delay resistance to EGFR-TKI, thus prolonging antitumor activity (Byers and Heymach, 2007; Le et al., 2021).

Subgroup analyses showed that the PFS benefit was consistently observed in EGFR-mutant advanced NSCLC patients of different gender (male or female), patients with different ECOG PS (0 or 1), baseline CNS metastasis (presence or absence) and EGFR mutation type (19del or 21L858R). The finding is echoed with the same subgroup analyses in the study of Deng et al., 2021. In addition, we found that combination bevacizumab and EGFR-TKI therapy significantly improved the PFS and OS result in smokers rather than those who never smoked, which is in line with the findings of Dafni et al., 2022. One possible explanation of this phenomenon is that TP53 mutation triggered by cigarette exposure would lead to increased sensitivity to anti-VEGF therapy (Schwaederlé et al., 2015). Moreover, we also noticed a significantly improved PFS in patients younger than 75 years old, as compared with those aged equal or more than 75 years. This finding is contradictory to that of Deng et al. (Deng et al., 2021). Nevertheless, it should be noted that the sample size of patients who aged equal or more than 75 years were too small in the ARTEMIS-CTONG1509 study and the PFS of the population could not be calculated, the number of patients aged equal or more than 75 years included was much less than those aged less than 75 years. In terms of different types of EGFR-TKI, our work included trials using all three generations of EGFR-TKI. Our data found that patients treated with erlotinib and bevacizumab combination therapy resulted in significantly better PFS than monotherapy, whereas the regimen involving osimertinib did not. The result may be partially explained by the fact that osimertinib and bevacizumab combination therapy adopted in both BOOSTER and WJOG8715L trials were used as non-first-line treatment. In the WJOG9717L trial, in which osimertinib and bevacizumab combination therapy was used in un-treated EGFR-mutant advanced NSCLC patients, bevacizumab was administered with a median duration of 33.4 weeks, which is shorter than that used with erlotinib (11–12 months) (Kenmotsu et al., 2022). There is another clinical trial (NCT04181060) currently evaluating the efficacy of osimertinib and bevacizumab combination therapy in un-treated EGFR-mutant advanced NSCLC patients, and the results are anticipated. Currently, most published work had focused on the Asian population. Our data showed that Asian population experienced significantly prolonged PFS than the non-Asian group. However, it should be noted that the sample size of non-Asian population is limited. There are several ongoing RCTs of EGFR-TKI with or without bevacizumab in EGFR-mutant advanced NSCLC that had primarily included non-Asian population (NCT04181060, NCT02971501), and the results are anticipated.

Noteworthy, several studies aimed to investigate the clinical value of multi-drugs therapy in treatment-naïve EGFR-mutant advanced NSCLC patients. The recently published FLAURA2 study confirmed significantly prolonged PFS in EGFR-mutant advanced NSCLC patients treated with osimertinib and chemotherapy, as compared with osimertinib alone (median PFS 25.5 months vs. 16.7 months, HR = 0.62, *p* < 0.001) (Planchard et al., 2023). The MARIPOSA study proved the superiority of amivantamab (with dual activity against EGFR and MET) and Lazertinib (a third-generation EGFR-TKI with CNS permeability) combination therapy in un-treated EGFR-mutant advanced NSCLC patients, as compared with osimertinib alone (median PFS 23.7 months vs. 16.6 months, HR = 0.7, *p* < 0.001) (Soria et al., 2013). The updated median PFS of the osimertinib monotherapy arm in the WJO9717L study was 20.2 months, which is longer than that reported in the FLAURA2 and MARIPOSA study. The reason may be that both FLAURA2 and MARIPOSA study had included more patients with CNS metastasis at baseline. With the emerging evidence of various combination therapy, the optimal choice for EGFR-mutant advanced NSCLC patients awaits further exploration.

However, the increased risk of combination therapy is non-neglectable. The most frequently observed grade≥3 TRAEs were hypertension, proteinuria, thrombotic events, rash, diarrhea and increased aminotransferase, which were similar to the established profiles of bevacizumab and EGFR-TKI, with no new safety concerns. Though it had been reported that the adverse effects of combination therapy were manageable (Kato et al., 2018), combination therapy of bevacizumab and EGFR-TKI should be applied with caution, and the occurrence of adverse events should be monitored carefully.

To the best of our knowledge, this is the meta-analysis that had included the most recently published RCTs comparing the clinical efficacy of combination therapy of bevacizumab and EGFR-TKI with EGFR-TKI monotherapy, and it is also the first meta-analysis that had performed subgroup analyses for both PFS and OS outcomes. However, some limitations should be taken under consideration. First, the majority of included trials had only involved Asian patients, and the non-Asian population is limited, which may affect the subgroup comparison between Asian group and non-Asian group. Second, the OS data of the AfaBev-CS study is immature and the subgroup analyses result are not reported, thus we failed to include the information in our work.

## Conclusion

Addition of bevacizumab to EGFR-TKI therapy provided significantly better PFS and ORR results for NSCLC patients harboring EGFR mutations, but no obvious OS benefit was observed and the risk of grade≥3 AEs was higher. Patients who ever smoked, aged <75 years old, and the Asian population might benefit more from the combination regimen, whereas gender, ECOG PS, baseline CNS metastasis and EGFR mutation type did not lead to significant differences.

## Data Availability

The raw data supporting the conclusion of this article will be made available by the authors, without undue reservation.
